# Clinical recurrence and antiplatelet drug resistance among patients with lower limb ischemia

**DOI:** 10.1097/MD.0000000000036915

**Published:** 2024-01-12

**Authors:** Nasr Alrabadi, Qusai Aljarrah, Osama Alzoubi, Hussam Al-Jarrah, Yasmin Elayyan, Zaid Alnabulsi, Anas Husein, Karem H. Alzoubi, Sohail Bakkar, Mukhallad Aljanabi, Malik Zihlif, Razan Haddad

**Affiliations:** a Department of Pharmacology, Faculty of Medicine, Jordan University of Science and Technology, Irbid, Jordan; b Department of Surgery, Faculty of Medicine, Jordan University of Science and Technology, Irbid, Jordan; c Department of Medicine, Division of Rheumatology, the University of Illinois, Chicago, IL, USA; d Department of Medicine, Beaumont Hospital, Royal Oak, MI, USA; e Department of Pharmacy Practice and Pharmacotherapeutics, University of Sharjah, Sharjah, United Arab Emirates; f Department of Clinical Pharmacy, Faculty of Pharmacy, Jordan University of Science and Technology, Irbid, Jordan; g Department of General and Specialized Surgery, Faculty of Medicine, the Hashemite University, Zarqa, Jordan; h Department of Physiology, Faculty of Medicine, Jordan University of Science and Technology, Irbid, Jordan; i Department of Pharmacology, School of Medicine, University of Jordan, Amman, Jordan; j Department of Pharmaceutical Sciences, Faculty of Pharmacy, Jadara University, Irbid, Jordan.

**Keywords:** aspirin, clopidogrel, PAD, platelet aggregation test, recurrence, resistance

## Abstract

There is a high prevalence rate of peripheral artery disease worldwide, with estimated cases exceeding 200 million. Most patients are under-diagnosed and under-treated, and there is a lack of knowledge regarding the best therapeutic regimen and therapy duration, which leads to many cases of recurrence, complications, and amputations. This study aims to explore clinical recurrence, which was defined as the worsening of chronic peripheral artery disease requiring hospital admission, and its relationship with antiplatelet drug resistance among patients with lower limb ischemia. This cohort study includes both retrospective and prospective recruitment of patients with chronic lower limb ischemia. Platelet aggregation tests were offered to the patients. Between February 2018 and November 2020, 147 patients were recruited from King Abdullah University Hospital and followed up for at least 1 year. Platelet aggregation tests were done for 93 patients who agreed to participate in this part of the study. The prevalence of chronic lower limb ischemia was higher in young male patients who are current smokers with co-morbid diseases such as hypertension, diabetes mellitus, and/or dyslipidemia. There was a significant association only of clinical recurrence with younger age (*P* = .011) and with low platelets count in severe stages of the disease (*P* = .047). No significant association was found in terms of laboratory resistance. The clinical recurrence rates of chronic lower limb ischemia were higher in younger patients and among those with low platelet counts in the severe stages of the disease. Despite the laboratory responsiveness to anti-platelet therapy, we observed significant clinical resistance and increased recurrence rates.

## 1. Introduction

Atherosclerosis is a lethal disease that affects the arteries where immune cells, lipids, and other elements accumulate and cause artery stenosis or occlusion.^[1]^ Atherosclerosis in the arteries of the extremities can result in peripheral artery disease (PAD), which occurs more often in the lower extremities.^[2]^ The prevalence of PAD is increased in elderly men who are obese, have a history of smoking, diabetes mellitus (DM), hypertension (HTN), a family history of premature atherosclerosis, chronic kidney disease, hypercholesterolemia or high fibrinogen concentration.^[3]^ On arrival at the clinic, patients with PAD demonstrate varying degrees of disease severity such as intermittent claudication, critical limb ischemia, ulcerations, and tissue necrosis ending with amputation, nevertheless, some patients might be asymptomatic.^[4]^

PAD patients are classified into different stages according to the severity of the symptoms using the Rutherford-Baker classification. Rutherford stage-0 patients are asymptomatic PAD patients, Rutherford stage-1 patients have mild claudication, Rutherford stage-2 patients have moderate claudication, Rutherford stage-3 patients have severe claudication, Rutherford stage-4 patients develop pain symptoms at rest, Rutherford stage-5 patients have minor tissue loss such as ulcers, and Rutherford stage-6 patients have major tissue loss.^[5]^ The increased platelet activation aggravates the severity of PAD, therefore, most of the available data support the use of anti-platelet therapy like aspirin (ASA) or clopidogrel in PAD patients, including asymptomatic Rutherford-Baker stage 0 patients.^[6,7]^

A previous study by Howard et al explored the occurrence of ASA resistance in patients with cardiovascular, cerebrovascular, and peripheral vascular disease. Their findings revealed that a considerable proportion of patients (up to 45%) may exhibit a variable response to ASA, resulting in insufficient inhibition of platelet function, a phenomenon defined broadly as ASA resistance.^[8]^ The exact mechanism of antiplatelet resistance is not well established. In a review conducted by Mărginean et al, various proposed mechanisms of such resistance were studied, including reduced bioavailability of antiplatelet drugs, genetic polymorphisms (especially in the P1A1/A2 polymorphism of the glycoprotein IIIa receptor gene and cytochrome P450 2C19 gene), alternative pathways of platelet activation, and accelerated platelet turnover as seen in cases of bleeding or stress.^[9]^ The review also provided a comprehensive discussion of the different mechanisms of antiplatelet drug resistance in critically ill patients. While some studies suggested that insufficient response to ASA could be due to the upregulation of cycloxegenase-2 and increased thromboxane production, a study by Zimmermann et al found that resistance is primarily driven by ASA inability to directly inhibit COX-1-mediated thromboxane synthesis in susceptible patients. This finding was further supported by similar results using supraphysiologic doses of ASA in vitro to inhibit cycloxegenase-1 in plasma concentrates extracted from the same patients.^[10–12]^ Another study conducted by Eikelboom et al also arrived at a comparable result, stating that the inclusion of clopidogrel does not impact the laboratory measurements of ASA inhibitory effects on platelet aggregation induced by arachidonic acid or urinary thromboxane.^[13]^

Previous studies have explored the presence of laboratory resistance for both ASA and clopidogrel among the Jordanian population without investigating the presence or the absence of clinical resistance.^[14–16]^ A previous study demonstrated a high prevalence of laboratory nonresponse to dual antiaggregatory medications in patients with critical limb ischemia. Nonresponse to ASA was found in 22% of cases, and nonresponse to adenosine-diphosphate (ADP)-inhibiting agents was observed in 47% of cases. Additionally, 14% of patients exhibited a concurrent absence of response to both agents.^[17]^ The MIRROR study investigated the occurrence of resistance in patients treated with dual antiplatelet therapy consisting of clopidogrel and ASA before and after endovascular therapy. The study specifically focused on clopidogrel resistance and found that approximately 30% of patients in the dual antiplatelet therapy group were resistant to clopidogrel. Among the 40 patients treated with dual antiplatelet therapy, 2 patients required revascularization through target lesion revascularization, and interestingly, both patients were among the clopidogrel-resistant individuals.^[18]^

In this study, we explore the laboratory resistance of clopidogrel and ASA determined by platelet aggregation analysis using the MULTIPLATE analyzer, in addition to the clinical resistance determined by the recurrence of PAD, which was defined as the worsening of chronic PAD requiring hospital admission during at least 1 year of follow-up. Furthermore, we explore whether laboratory resistance is associated with clinical recurrence, and we study clinical and laboratory factors that could be associated with clinical recurrence.

## 2. Methods

### 2.1. Patients’ population

This cohort study includes both retrospective and prospective recruitment of chronic PAD patients (patients with chronic lower limb ischemia) from King Abdullah University Hospital at Jordan University of Science and Technology between February 2018 and November 2020 with at least 1 year of follow-up. The recruitment of the patients stopped within the first wave of the COVID-19 pandemic in Jordan and patients with COVID-19 infection or vaccination were excluded from the study. All patients older than 18, being males or females, with a confirmed diagnosis of chronic lower limb ischemia from a vascular specialist who was recruited for the aim of this study. Both regular hospital visits and urgent hospital admission were included. Patients were enrolled after signing an informed consent form in accordance with the Institutional Review Board. Patients with traumatic ischemia, vasculitis-induced ischemia, ischemia related to non-atherosclerotic causes, and isolated acute limb ischemia were excluded from the study.

### 2.2. Patient assessment and blood sample collection

Patients’ demographics, co-morbidities, medication history, laboratory readings such as platelet count, hemoglobin level, glycated hemoglobin level, estimated glomerular filtration rate, international normalized ratio, creatinine and other medical comorbidities such as HTN, DM, ischemic heart diseases, stroke, chronic kidney disease were collected from the hospital medical electronic system, in addition to previous lower limb ischemic events (regular visits or acute presentations). Clinical recurrence was defined as worsening of chronic PAD requiring hospital admission during at least a year of follow-up.

### 2.3. Method of performing the MULTIPLATE test

All procedures were conducted according to the manufacturer recommendations for the multiplate analyzer system (Verum Diagnostica GmbH, Munich, Germany) and kits that were used (HART-UK). Briefly, within a maximum of 15 minutes of drawing the samples, a small amount of whole blood (0.3 mL per test) is applied into the Multiplate channel. Each test takes place in a single-use test cell, which incorporates a dual sensor unit and a Teflon-coated stirring magnet. The principle of Multiplate analysis is based on the fact that platelets get sticky upon activation by adding the following agents: arachidonic acid (Cat#: HB-5506-FG), Collagen (Cat #: HB-S504-FG), Thrombin Receptor Activator for Peptide 6 (Cat #: HB-5512-FG), ADP (Cat#: HB-5502-FG), and Ristocetin (Cat #: HB-5508-FG), which leads to platelet aggregate on metal sensor wires in the Multiplate test cell. Multiplate sensors are transformed into arbitrary aggregation units and plotted against time. In concordance with previous studies, laboratory resistance to ASA or clopidogrel was defined as a reduction of more or equal to 30% of the normal reference range given by the machine manufacturer (MULTIPLATE Analyzer), in which the ADP test values reflected the degree of clopidogrel responsiveness while arachidonic-acid-induced platelet aggregation (ASPI) test values reflected the degree of ASA responsiveness.

### 2.4. Statistical analysis

Data were analyzed using SPSS Version 26. Categorical variables were represented as a number (percentage). A multivariate logistic regression model was created having the clinical recurrence as the outcome variable, while demographic characteristics, laboratory parameters, and platelet aggregation studies were considered predictor variables. All predictor variables were adjusted by factors of age, smoking status, and gender. We recurrently assessed the goodness-of-fit of all regression models through the Hosmor and Lemeshow test. A significance level of < 0.05 was used. Clinical recurrence was defined as the recurrence of PAD during a follow-up period of at least 1 year. Patients were divided into a PAD group with gangrene/tissue loss (Rutherford classification from R5-R6) and a PAD group without gangrene/tissue loss (Rutherford classification from R0-R4) in which each group was explored separately.

## 3. Results

### 3.1. Patients demographics

Table 1 shows demographic data, baseline laboratory data, and clinical characteristics for all 147 patients. Ninety-three patients agreed to provide blood samples dedicated to platelet aggregation testing. Overall, 82.9% of our sample were males (122/147). Overall, 55.1% had a Rutherford PAD classification of (R0–R4) which was classified as the PAD group without gangrene/tissue loss, while 44.9% had a Rutherford PAD classification of (R5–R6) which was classified as the PAD group with gangrene/tissue loss. The majority (53.1%) of the population were on dual antiplatelet treatment with both ASA and clopidogrel, while 29.9% and 10.2% were treated with ASA and clopidogrel alone respectively. Table 2 illustrates the percentages of clinical recurrence and laboratory resistance among the study sample, stratified according to the antiplatelet regimen and PAD severity class. No significant differences between males and females were detected in terms of clinical recurrence or laboratory resistance (*P* > .05).

**Table 1:** General characteristics and demographics of the study participants.

Characteristics	Overall (N = 147)	Patients whom aggregation tests have been obtained (N = 93)
Gender		
Male	122 (82.9%)	72 (77.4%)
Female	25 (17%)	21 (22.6%)
Smoker		
Not smoker	69 (46.9%)	37 (39.8%)
Current smoker	63 (42.9%)	45 (48.4%)
Ex-smoker	15 (10.2%)	11 (11.8%)
Class		
(PAD group without gangrene/tissue loss) R0 + R1 + R2 + R3 + R4	81 (55.1%)	46 (49.5%)
(PAD group with gangrene/tissue loss) R5 and R6	66 (44.9%)	47 (50.5%)
Recurrence R0–R4		
Yes	40 (49.4%)	23 (50.0%)
No	41 (50.6%)	23 (50.0%)
Recurrence R5 and R6		
Yes	38 (57.6%)	25 (53.2%)
No	28 (42.4%)	22 (46.8%)
DM		
Yes	116 (78.9%)	75 (80.6%)
No	31 (21%)	18 (19.4%)
Dyslipidemia		
Yes	87 (59.2%)	65 (70.0%)
No	60 (40.8%)	28 (30.0%)
HTN		
Yes	51 (34.7%)	61 (65.6%)
No	96 (65.3%)	32 (34.4%)
Anti-platelet		
ASA	44 (29.9%)	28 (30.1%)
Clopidogrel	15 (10.2%)	8 (8.6%)
Both	78 (53.1%)	57 (61.3%)
Nothing	10 (6.8%)	0 (0.0%)

Ex-smoker: someone who has smoked more than 100 cigarettes in their lifetime but has not smoked in the last 28 d.

Defined as having fasting blood glucose values ≥ 126 mg/dL, or 2-h plasma glucose values of ≥ 200 mg/dL during a 75 g oral glucose tolerance test (OGTT), or HbA1c values ≥ 6.5%.

Dyslipidemia: Defined as having abnormally high total cholesterol (TC), low-density lipoprotein cholesterol (LDL-C), non-high-density lipoprotein cholesterol (non-HDL-C), or triglycerides above laboratory-specific range. Or abnormally low HDL-C below laboratory-specific range.

Defined as having an abnormally elevated systolic blood pressure of equal or >130 mm Hg or diastolic blood pressure of equal or >80.

ASA = Aspirin, DM = diabetes mellitus, HTN = hypertension, PAD = peripheral arterial disease.

**Table 2:** Clinical recurrence and laboratory resistance of the study sample, stratified according to the antiplatelet regimen and PAD severity class into PAD group without gangrene/tissue loss (R1–R4) and PAD group without gangrene/tissue loss (R5–R6).

	Clinical Recurrence (%)	Laboratory resistance* (%)
R0–R4 PAD patients taking aspirin alone	62.5% (15/24)	22.2% (2/9)
R0–R4 PAD patients taking clopidogrel alone	28.6% (2/7)	0% (0/1)
R0–R4 PAD patients taking dual antiplatelet therapy (aspirin + clopidogrel)	46.6% (21/45)	16.6% (4/24) resistance to aspirin and 4.2% (1/24) resistance to clopidogrel
R5–R6 PAD patients taking aspirin alone	60.0% (12/20)	21.4% (3/14)
R5–R6 PAD patients taking clopidogrel alone	50.0% (4/8)	66.7% (4/6)
R5–R6 PAD patients taking dual antiplatelet therapy (aspirin + clopidogrel)	57.6% (19/33)	50.0% (10/20) resistance to aspirin and 35.0% (7/20) resistance to clopidogrel

* Defined as a reduction of more or equal to 30% of the normal reference range of ASPI and ADP tests for patients taking Aspirin and/or Clopidogrel respectively.

PAD = peripheral arterial disease.

### 3.2. Predictors of clinical recurrence among PAD patients

The output of logistic regression for R1, R2, R3, and R4 patients (n = 81) after adjustment according to age, gender, and smoking status to prevent the cofounder effect is shown in Table 3. None of the studied variables including baseline laboratory data, comorbidities, and antiplatelet aggregation tests have significantly correlated with recurrence (Table 3).

**Table 3:** Multivariate logistic regression model output of recurrence predictors for R0, R1, R2, R3, and R4 patients.

Predictor (n)	Adjusted odds ratio				
	Correlation coefficient	Standard error	*P* value	Odds ratio	95% C.I.
Creatinine (81)	−0.001	0.002	.806	0.999	0.995–1.004
DM (81)	0.122	0.524	.817	1.129	0.404–3.153
Dyslipidemia (81)	−0.061	0.889	.946	0.941	0.165–5.373
Age (81)	−0.047	0.023	.737	0.746	0.135–4.115
eGFR (81)	−0.010	0.007	.163	0.990	0.976–1.004
Hb (81)	0.128	0.102	.211	1.136	0.930–1.388
HbA1c (81)	0.053	0.123	.666	1.055	0.828–1.343
Platelet count (81)	−0.003	0.002	.184	0.997	0.992–1.001
HTN (81)	0.234	0.844	.782	1.264	0.242–6.611
IHD (81)	0.406	0.483	.401	1.500	0.582–3.870
INR (81)	0.001	1.390	1.000	1.001	0.066–15.243
Aspirin use (81)	0.881	0.713	.217	2.412	0.596–9.761
Clopidogrel use (81)	−0.471	0.494	.341	0.625	0.237–1.644
PPI use (81)	0.347	0.513	.499	1.414	0.517–3.866
Statin use (81)	0.096	0.481	.842	1.101	0.429–2.823
Use of clopidogrel with aspirin (compared to aspirin alone) (70)	0.717	0.548	.191	2.048	0.700–5.994
ASPI test (33)	0.005	0.011	.650	1.005	0.983–1.028
Aspirin laboratory responsiveness (ASPI test—binary) (33)	0.211	1.053	.841	1.235	0.157–9.727
Use of aspirin with clopidogrel (compared to clopidogrel alone) (52)	0.719	0.932	.441	2.052	0.330–12.761
ADP test (25)	−0.023	0.028	.415	0.977	0.924–1.033

ADP = adenosine diphosphate aggregometry test, ASPI = Multiplate ASPItest aggregometry, DM = diabetes mellitus, eGFR = estimated glomerular filtration rate, Hb = hemoglobin, HbA1c = glycated hemoglobin, HTN = hypertension, IHD = ischemic heart disease, INR = international normalized ratio, PPI = proton pump inhibitor.

The outputs of logistic regression for R6 and R5 patients (n = 66) after adjustment according to age, gender, and smoking status to prevent the cofounder effect are shown in Table 4. Age was significantly inversely correlated with recurrence (*P* = .011) with a correlation coefficient of (−0.082). Furthermore, platelet count also was significantly inversely correlated with recurrence (*P* = .047) with a correlation coefficient of (−0.006). Other variables studied did not exhibit a significant correlation with recurrence (Table 4).

**Table 4:** Multivariate logistic regression model output of recurrence predictors for R5 and R6 patients. Significant predictors are *italicized*.

Predictor	Adjusted odds ratio				
	B	S.E	Sig.	Odds ratio	95% C.I.
Creatinine (66)	0.002	0.002	0.449	1.002	0.997–1.006
DM (66)	1.075	1.052	0.307	2.931	0.373–23.059
Dyslipidemia (66)	0.788	0.772	0.307	2.199	0.484–9.983
*Age* (66)	*−0.082*	*0.032*	*0.011*	*0.921*	*0.864–0.982*
eGFR (66)	0.000	0.007	0.989	1.000	0.987–1.013
Hb (66)	0.077	0.108	0.473	1.081	0.875–1.335
HbA1c (66)	0.071	0.138	0.605	1.074	0.820–1.407
*Platelet count* (66)	−*0.006*	*0.003*	*0.047*	*0.994*	*0.988–0.999*
HTN (66)	0.552	0.950	0.561	1.737	0.270–11.176
IHD (66)	0.103	0.590	0.861	1.109	0.349–3.522
INR (66)	0.604	0.814	0.458	1.829	0.371–9.018
Aspirin use (66)	0.092	0.673	0.892	1.096	0.293–4.102
Clopidogrel use (66)	0.007	0.564	0.990	1.007	0.334–3.040
PPI use (66)	0.512	0.629	0.416	1.668	0.486–5.722
Statin use (66)	0.737	0.563	0.191	2.089	0.693–6.297
Use of clopidogrel with aspirin (compared to aspirin alone) (53)	−0.291	0.660	0.659	0.747	0.205–2.727
ASPI test (34)	−0.001	0.010	0.919	0.999	0.979–1.019
Aspirin laboratory responsiveness (ASPI test—binary) (34)	0.108	0.944	0.909	1.114	0.175–7.091
Use of aspirin with clopidogrel (compared to clopidogrel alone) (41)	0.137	0.951	0.886	1.147	0.178–7.393
ADP test (26)	−0.007	0.013	0.600	0.993	0.967–1.019
Clopidogrel laboratory responsiveness (ADP test—binary) (26)	0.649	1.017	0.523	1.913	0.261–14.032

ADP = adenosine diphosphate aggregometry test, ASPI = Multiplate ASPItest aggregometry, DM = diabetes mellitus, eGFR = estimated glomerular filtration rate, Hb = hemoglobin, HbA1c = glycated hemoglobin, HTN = hypertension, IHD = ischemic heart disease, INR = international normalized ratio, PPI = proton pump inhibitor.

## 4. Discussion

The average age for the patients recruited in this study was 60.8 years old, similar to other studies and corresponding to the increasing prevalence of PAD as age increases.^[19,20]^ Our study found a significant association between recurrence and age, in which recurrence was inversely related to age. This means that younger patients in our sample experienced higher recurrence rates than older patients. Such predilection for younger age groups may be explained by the finding that younger patients are more likely to have inherited hypercoagulable disorders, such as essential thrombocytosis. However, our study did not include specific diagnostic evaluations for such disorders.^[21]^ Also, in this context, a previous study showed higher rates of early re-thrombosis and major amputations in younger patients who failed thrombolytic therapy when compared to older patients, which was attributed to the higher smoking rates in younger patients.^[22]^ Furthermore, it is well known that atherosclerosis has such a virulent course with high rates of recurrence or progressive disease which is strikingly associated with cigarette smoking.^[23]^

Cigarette smoking contributes to the recurrence of ischemic events, anti-platelet resistance, and high PAD prevalence by causing a shift in the homeostasis balance toward thrombus formation by causing endothelial injury, plaques formation, plaques rupture, platelet activation, platelet aggregation and platelet adhesion stimulating coagulation cascade.^[24]^ One study showed that ASA was unable to affect the increased aggregation even after smoking a single cigarette,^[25]^ furthermore increased urinary excretion of thromboxane A2 in habitual smokers was demonstrated in another study.^[26]^ Cigarette components, which are nicotine and polycyclic aromatic hydrocarbons, affect clopidogrel metabolism by increasing the activity of cytochrome P450 1A2 hepatic enzymes, which might explain why most clopidogrel responders were smokers^[27]^; thus, doubling the clopidogrel dose of nonsmokers might be needed.^[28]^ However, in our study, we did not find a significant association between smoking status and recurrence rate. This could be due to the limitation of smoking-related data collection, where patients were classified as current, previous, or nonsmokers without considering the qualitative pack-years status.

Another interesting finding in our study was the significant association between recurrence rates and lower platelet levels among patients with stage 5 and 6 PAD (R5, R6 according to Rutherford classification). This laboratory predictor for recurrence was previously highlighted in other studies, where platelet count was lower in comparison to the healthy control group but without sufficient explanations.^[29,30]^ Another study reported that an increased ratio of mean platelet volume to platelet count was a predictor for re-occlusion in Coronary artery bypass graft surgery patients.^[31]^ Nevertheless, this might be due to inflammatory or genetic factors that affect platelets and blood components during more severe stages of PAD. Further studies are needed to reach a plausible explanation.

Platelet aggregation tests include the ADP test (measures responsiveness to clopidogrel), and the ASPI test (measures responsiveness to ASA). Low values (lower than normal reference range) indicate a normal decrease in platelet aggregation as a response to ASA or clopidogrel, i.e., laboratory responsiveness. Our study also explored the relationship between laboratory responsiveness as resembled by ADP and ASPI test values, and recurrence rates. Our results didn’t show any significant association between ADP and ASPI test values and recurrence, which might indicate that clinical recurrence is a multifactorial process with combined factors including (age, genetics, coagulation, blood products, drug-drug interactions, pharmacokinetics…, etc) rather than being only related to the pharmacodynamic aspect of drug responsiveness as indicated by successful inhibition of platelet aggregation. This is also seen in other studies, where ASA laboratory resistance is not a predictor of outcome in patients after coronary stenting.^[32]^

Our results also showed no significant difference in the recurrence of lower limb ischemia between males and females. A previous study concluded that ASA insensitivity is much less common in males and more common in patients with kidney problems. Such a discrepancy suggests again that other factors may play a significant role in the recurrence of peripheral vascular diseases.^[33]^

No significant association was found between the use of ASA or clopidogrel or both and the recurrence rates of ischemia in PAD. This is why we need to evaluate other factors that participate in PAD recurrence such as the delay in diagnosis of PAD,^[34]^ inflammation, which may cause atherosclerotic plaque rupture and high rates of platelets turnover,^[35]^ poor patient adherence to medications,^[36]^ PAD severity, DM, HTN, genetic polymorphisms, and dyslipidemia.^[37]^

Our study was limited by its relatively small sample size which may have impacted the power of the study and hindered the utility of more flexible analytic methods. However, the controlling and fitness assessments along with the exploratory nature of our study guide future wider validation studies and contributed to this field. Furthermore, some aspects of data collection may have impacted our results, most importantly in the aspect of smoking status, where the lack of quantitative assessment (i.e., pack year) may have led to insignificant results by statistical analysis.

To conclude, we need to adopt a new hypothesis to describe PAD pathophysiology that no longer frames platelet aggregation as a cornerstone. This reinforces recent studies that considered additional drug classes like anticoagulants, including the COMPASS Trial which found that a combination of rivaroxaban 2.5 mg twice daily plus ASA was more favorable than ASA monotherapy.^[38]^ Our results also advocated toward focusing more on the inflammatory process and the most dominant blood biomarkers in the late stages of PAD to facilitate targeting and monitoring them in the early stages of the disease.

## Acknowledgments

We acknowledge the role and the help of Dr Asma Alshhobi and Mrs. Alaa Magd while conducting the study and recruiting patients.

## Author contributions

**Conceptualization:** Nasr Alrabadi, Qusai Aljarrah, Osama Alzoubi, Hussam Al-Jarrah, Yasmin Elayyan, Zaid Alnabuls, Karem H. Alzoubi, Sohail Bakkar, Mukhallad Aljanabi, Malik Zihlif, Razan Haddad.

**Data curation:** Nasr Alrabadi, Qusai Aljarrah, Hussam Al-Jarrah, Yasmin Elayyan, Zaid Alnabuls, Karem H. Alzoubi, Sohail Bakkar, Mukhallad Aljanabi, Malik Zihlif, Razan Haddad.

**Formal analysis:** Nasr Alrabadi, Qusai Aljarrah, Osama Alzoubi, Hussam Al-Jarrah, Yasmin Elayyan, Mukhallad Aljanabi, Razan Haddad.

**Funding acquisition:** Nasr Alrabadi.

**Investigation:** Nasr Alrabadi, Qusai Aljarrah, Osama Alzoubi, Hussam Al-Jarrah, Yasmin Elayyan, Zaid Alnabuls, Karem H. Alzoubi, Sohail Bakkar, Mukhallad Aljanabi, Malik Zihlif, Razan Haddad.

**Methodology:** Nasr Alrabadi, Qusai Aljarrah, Osama Alzoubi, Hussam Al-Jarrah, Yasmin Elayyan, Zaid Alnabuls, Anas Husein, Sohail Bakkar, Mukhallad Aljanabi, Malik Zihlif.

**Project administration:** Nasr Alrabadi, Qusai Aljarrah, Karem H. Alzoubi, Mukhallad Aljanabi, Malik Zihlif.

**Resources:** Nasr Alrabadi, Qusai Aljarrah, Karem H. Alzoubi, Mukhallad Aljanabi, Malik Zihlif.

**Supervision:** Nasr Alrabadi, Razan Haddad.

**Validation:** Nasr Alrabadi, Karem H. Alzoubi, Razan Haddad.

**Visualization:** Nasr Alrabadi, Qusai Aljarrah, Osama Alzoubi, Yasmin Elayyan, Zaid Alnabuls, Anas Husein, Karem H. Alzoubi, Sohail Bakkar, Mukhallad Aljanabi, Malik Zihlif, Razan Haddad.

**Writing – original draft:** Nasr Alrabadi, Osama Alzoubi, Hussam Al-Jarrah, Yasmin Elayyan, Zaid Alnabuls, Anas Husein, Sohail Bakkar, Mukhallad Aljanabi, Malik Zihlif, Razan Haddad.

**Writing – review & editing:** Nasr Alrabadi, Karem H. Alzoubi.
